# Standardization of platelet releasate products for clinical applications in cell therapy: a mathematical approach

**DOI:** 10.1186/s12967-017-1210-z

**Published:** 2017-05-19

**Authors:** Francesco Agostini, Jerry Polesel, Monica Battiston, Elisabetta Lombardi, Stefania Zanolin, Alessandro Da Ponte, Giuseppe Astori, Cristina Durante, Mario Mazzucato

**Affiliations:** 1 Stem Cell Unit, CRO Aviano National Cancer Institute, Aviano, PN Italy; 2 Unit of Cancer Epidemiology, CRO Aviano National Cancer Institute, Aviano, PN Italy; 3 Advanced Cellular Therapy Laboratory, Department of Cellular Therapy and Hematology, San Bortolo Hospital, Vicenza, Italy

**Keywords:** Growth factors from human platelets, Releasate standardization, Mathematical algorithm, Cell therapy, Good manufacturing practice, ASC ex vivo expansion

## Abstract

**Background:**

Standardized animal-free components are required for manufacturing cell-based medicinal products. Human platelet concentrates are sources of growth factors for cell expansion but such products are characterized by undesired variability. Pooling together single-donor products improves consistency, but the minimal pool sample size was never determined.

**Methods:**

Supernatant rich in growth factors (SRGF) derived from n = 44 single-donor platelet-apheresis was obtained by CaCl_2_ addition. n = 10 growth factor concentrations were measured. The data matrix was analyzed by a novel statistical algorithm programmed to create 500 groups of random data from single-donor SRGF and to repeat this task increasing group statistical sample size from n = 2 to n = 20. Thereafter, in created groups (n = 9500), the software calculated means for each growth factor and, matching groups with the same sample size, the software retrieved the percent coefficient of variation (CV) between calculated means. A 20% CV was defined as threshold. For validation, we assessed the CV of concentrations measured in n = 10 pools manufactured according to algorithm results. Finally, we compared growth rate and differentiation potential of adipose-derived stromal/stem cells (ASC) expanded by separate SRGF pools.

**Results:**

Growth factor concentrations in single-donor SRGF were characterized by high variability (mean (pg/ml)–CV); VEGF: 950–81.4; FGF-b: 27–74.6; PDGF-AA: 7883–28.8; PDGF-AB: 107834–32.5; PDGF-BB: 11142–48.4; Endostatin: 305034–16.2; Angiostatin: 197284–32.9; TGF-β1: 68382–53.7; IGF-I: 70876–38.3; EGF: 2411–30.2). In silico performed analysis suggested that pooling n = 16 single-donor SRGF reduced CV below 20%. Concentrations measured in 10 pools of n = 16 single SRGF were not different from mean values measured in single SRGF, but the CV was reduced to or below the threshold. Separate SRGF pools failed to differently affect ASC growth rate (slope pool A = 0.6; R^2^ = 0.99; slope pool B = 0.7; R^2^ 0.99) or differentiation potential.

**Discussion:**

Results deriving from our algorithm and from validation utilizing real SRGF pools demonstrated that pooling n = 16 single-donor SRGF products can ameliorate variability of final growth factor concentrations. Different pools of n = 16 single donor SRGF displayed consitent capability to modulate growth and differentiation potential of expanded ASC. Increasing the pool size should not further improve product composition.

**Electronic supplementary material:**

The online version of this article (doi:10.1186/s12967-017-1210-z) contains supplementary material, which is available to authorized users.

## Background

Ex vivo cell expansion is often required to obtain advanced therapy medicinal products (ATMPs) for clinical use. As stated in the European Regulation 1394/2007, ATMPs must be obtained in compliance with good manufacturing practice (GMP) guidelines [1]. Employment of validated and standardized animal and xeno-free compounds for cell manufacturing process is crucially important to preserve clinical safety of the final product [2]. Fetal bovine serum is the most common source of growth factors, and it is routinely used in research protocols as culture medium additive to promote cell expansion. Nevertheless, fetal bovine serum contains xeno-carbohydrates and xeno-proteins potentially leading to undesired clinical effects upon ATMPs administration to patients [3–6]. Replacement of fetal bovine serum use is strongly recommended throughout Europe and United States [7, 8] for cell product manufacturing under GMP guidelines. Human platelet concentrates represent a suitable, abundant and cost effective alternative source of growth factors. In previous publications human growth factors, derived by application of repeated freeze and thaw cycles to platelet concentrates, were utilized to expand cells in vitro [9–14]. We previously published a method to obtain a supernatant rich in growth factors (SRGF) derived from platelet-apheresis concentrates by endogenous thrombin activation and subsequent triggering of coagulation cascade [15]. Nevertheless, final concentrations of growth factors obtained from single donor platelet concentrates are highly variable due to donor inter-individual differences [16, 17]. To comply with GMP guidelines [1], separate batches of medium additives must be characterized by consistent concentrations of growth factors in order to consistently promote ex vivo cell expansion. Pooling together several single donor preparations can minimize the final product variability [13, 14]; otherwise, the lowest number of single donor SRGF, needed for production of pool products characterized by sufficient consistency between batches, would still need to be assessed.

## Methods

### SRGF production

Single donor SRGF was obtained as described in our previous publication [15]. Briefly, a platelet rich plasma (PRP) obtained from single donor platelet apheresis product was added with CaCl_2_ (Monico, Venice, Italy) at the final concentration of 0.04 M and incubated at 40 °C for approximately 60 min. Supernatant was separated from clot by centrifugation at 1600×*g* for 15 min at room temperature. Aliquots were stored at −80 °C until analysis. Each single donor SRGF was labeled by a progressively growing identification code (from 1 to 44). Selection of the 44 blood donors was performed according to the Italian Laws and to the guidelines issued by the “Centro Nazionale Sangue” (Italian Ministry of Health). The mean donor age was 47 years (range from 23 to 58 years). None of the selected donors were taking drugs that could have potentially interfered with platelet function for at least 2 weeks before donation. Circulating platelet count of selected donors was >180,000 platelets/μl.

### Concentration measurements

Concentrations of vascular endothelial growth factor (VEGF), epidermal growth factor (EGF), platelet derived growth factor-AA, -AB, -BB (PDGF-AA, AB, -BB), transforming growth factor-β1 (TGF-β1), fibroblast growth factor basic (FGF-basic), insulin like growth factor-I (IGF-I) were measured utilizing Quantikine ELISA kits (R&D Systems, Minneapolis, MN, US). Concentrations of Endostatin (ES), Angiostatin (AS) were measured by RayBio ELISA kit (Raybiotech, Norcross, GA, US). Concentrations of growth factors were measured in 44 SRGF obtained from different single donors. Concentrations were also measured in small scale pool product batches (n = 10, from A to J). Each pool batch was created—according to algorithm derived results—mixing together 16/44 aliquots of randomly chosen single donor SRGF. To create SRGF pool batches C–J, 500 μl of each single-donor SRGF were utilized in a final volume of 8 ml. Since higher final volumes of SRGF pools A and B were required to perform cell proliferation assays, 2 ml of each single donor SRGF were mixed together in a final volume of 32 ml.

### Mathematical algorithm for pool size estimation

In order to mathematically predict the optimal pool size that reduces the variability of growth factors concentrations within an acceptable value, we designed a de novo statistical algorithm to simulate the creation of a pool using different number of specimens. The algorithm was developed using the R-software, an open source statistical software [18]. Single values of VEGF, FGF-basic, PDGF-AA, -AB, -BB, ES, AS, TGF-β1, IGF-I, EGF measured in 44 different specimens from single donors, were utilized as input data. For each SRGF product, the procedure randomly extracted 500 statistical samples of a defined sample size from the pool of 44 specimens using a bootstrap re-sampling method [19]. For each statistical sample the mean value of the SRGF product was firstly calculated and kept as the SGRF product concentration for the pool; then, mean value, standard deviation and percent coefficient of variation (CV) of concentrations were calculated over the 500 statistical samples. This operation was repeated for increasing sample sizes, starting from n = 2 to n = 20. Data were plotted as software output (Fig. 2). In order to choose the appropriate pool size, the CV threshold (20%; solid line in Fig. 2) was defined across the lowest estimated CV value (at horizontal asymptote) identified for the most variable growth factor (VEGF).

### Thrombin generation potential

To further improve characterization of SRGF composition, thrombin generation potential was assessed by Thrombin Generation Assay kit (Technoclone GmbH; Vienna, Austria) following manufacturer’s instruction. Low recombinant tissue factor (LTF) or high recombinant tissue factor (HTF) concentrations were added to SRGF samples or PRP from apheresis product (as reference and positive control) immediately before the assay. Thrombin activity kinetics were detected by monitoring changes over time of fluorescence generated by a fluorogenic substrate. Data acquisition was performed utilizing Infinite^®^ F200 (Tecan; Männedorf, Switzerland) as fluorescence reader. To determine non specific fluorescence values, coagulation cascade was activated in independent samples added with an excess (100 U/ml final concentration) of heparin (Epsoclar; Hospira S.r.l., Naples, Italy). Peak fluorescence values measured in heparin treated samples were lower than 10% of related values measured in PRP samples. Non specific fluorescence values were subtracted from fluorescence values measured in samples of PRP and of SRGF pool batches. Calculations of peak thrombin concentrations were performed by means of mathematical algorithms provided by the manufacturer.

### Cell cultures

To verify the consistency of different SRGF pools as additives in cell culture medium we compared the impact of both SRGF pool batch A and SRGF pool batch B on growth rate of adipose tissue-derived stromal cells (ASC) [20]. ASC were derived from stromal vascular fraction obtained by collagenase digestion of adipose tissue aliquots taken by liposuction from n = 2 female breast cancer patients (age: 52 and 54 years) that underwent mammary reconstruction by lipotransfer. The protocol was approved by the Ethics Committee of the CRO Aviano National Cancer Institute (Protocol Number: CRO-2016-30) and it complied with the Declaration of Helsinki (2004).

After digestion of washed lipoaspirate by collagenase (NB 6 good manufacturing practice grade, SERVA Electrophoresis GmbH, Heidelberg, Germany—0.15 U/ml final concentration), cell suspension was centrifuged (400×*g* for 10 min at +4 °C) and washed with a solution composed by 10% human albumine (Albital, Kedrion, Italy) 10% Acid Citrate Dextrose Solution-A (ACD-A; Haemonetics Corporation, Braintree, MA; USA), 2 U/ml heparin (Epsodilave, HOSPIRA ITALIA S.r.l., Napoli; Italy) in Ringer Lactate solution (Fresenius Kabi Italia, VR, Italy). Nucleated cells were seeded on vented tissue culture treated flasks (Falcon^®^-Corning NY; USA) at the density of 50,000 viable cells/cm^2^ in Minimum Essential Medium Eagle-Alpha Modification (Alpha-MEM, Lonza; Basel, Switzerland) added with 100 IU/ml of Penicillin and 100 μg/ml of Streptomycin (Sigma, St. Louis, MO; USA) and with 5% (vol/vol) SRGF pool—batch A or SRGF pool—batch B. Seeded cells were allowed to adhere for 24 h, then non-adherent cells were removed and fresh medium was added after a single wash with phosphate buffered saline (PBS) solution (Sigma). Growth of attached cells (P0) was daily monitored by phase contrast microscopy (Olympus CKX41, Olympus Italia Srl, Milano; Italy). Upon 80–90% confluence, cells were detached by trypsin (TrypLe Select 10×, Life Technologies, Carlsbad, CA, US). In order to perform cell proliferation assay in the same time period, washed cells were cryopreserved by resuspension in SRGF pool—batch A or SRGF pool—batch B added with 10% dimethyl sulfoxide (CryoSure-DMSO, Li StarFish, Milano, Italy). Cells were frozen in two steps: a transient freezing (overnight) at −80 °C in a container designed to allow a temperature decrease of −1 °C/min, (Mr Frosty, Thermo Scientific, Waltham, MA; USA) followed by final storage in liquid nitrogen vapor phase. After thawing, resuspended cells were seeded at passage 1 (P1) at the density of 1000–2000 cells/cm^2^. Each additional cell passage was performed in the same seeding conditions and population doublings (PD) were calculated as follows: PD at P_i_ = 3.32 × (log n2_i_ − log n1_i_); where n1_i_ is the number of seeded cells and n2_i_ is the number of harvested cells at selected passage (P_i_). Cumulative PD (cPD) was calculated as follows: cPD = PD at P_i−1_ + PD at P_i_. Population doubling time was calculated as follows: PDT_i_ = (tP_i_ − tP_i−1_) × 24/PD; (tP_i_ − tP_i−1_) × 24 is the time interval (hours) between consecutive cell passages.

### ASC differentiation potential assay

At P3-P4, part of ASC expanded in complete Alpha-MEM medium added with 5% (vol/vol) SRGF—batch A or SRGF—batch B were detached by trypsinization. Cells were seeded at 10,000 cells/cm^2^ and after complete adhesion (24 h) adipogenic, chondrogenic and osteogenic differentiation was induced utilizing StemMACS AdipoDiff, ChondroDiff and OsteoDiff media (Miltenyi Biotec GmbH, Bergisch Gladbach; Germany). After 21 days, differentiated osteocytes, adipocytes and chondrocytes were stained by Alizarin Red, Oil Red-O and Safranin-O (Sigma), respectively.

### Statistics

**Fig. 1:**
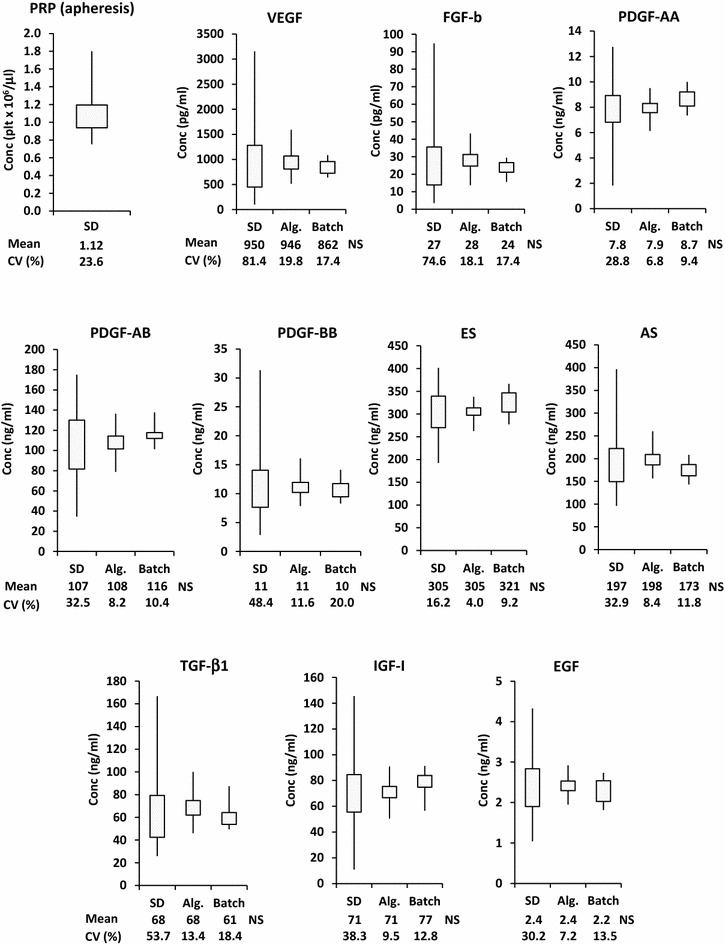
*Squared box plot* represents the distribution of platelet concentration (Conc.) values measured in single donor (SD) PRP, obtained from platelet apheresis product. The figure depicts distribution of growth factor concentration values measured in n = 44 SD SRGF specimens (*left box plots*).* Middle box plots* show the distribution of mean values calculated in algorithm-created (Alg.) groups (n = 500) of single donor SRGF concentration data (n = 16, in each group). *Right box plots* display distribution of growth factor concentrations measured in n = 10 real pools (Batch) each composed of n = 16 single donor SRGF specimens. Mean or grand mean values as well as coefficient of variation [CV (%)] are reported below each *box plot*. *NS* not significantly different vs Alg. and SD; ANOVA for independent samples

Significance of difference between means was tested by ANOVA for independent samples test (Fig. 1). Linearity of growth curves was tested calculating R^2^ as measure of goodness of fit of linear regression.

## Results

**Fig. 2:**
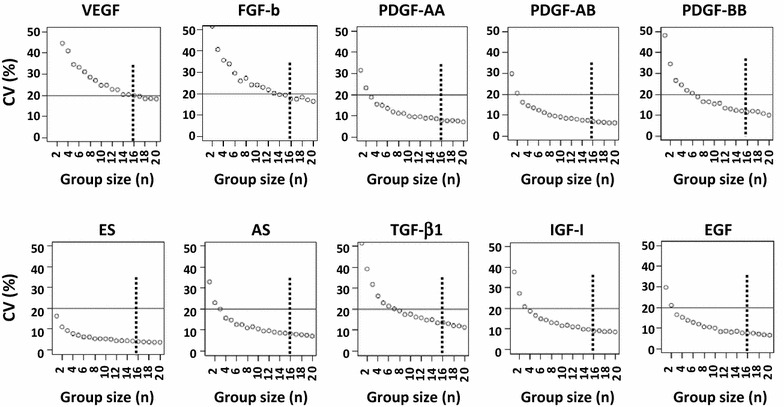
Graphical representation of output results obtained by calculations performed by the statistical algorithm. The algorithm was programmed to create 500 statistical groups of random data from single donor SRGF and to repeat this operation by increasing the group statistical sample size step by step from n = 2 to n = 20. Thereafter, in created groups (n = 9500), the software calculated means for each growth factor and, matching groups characterized by the same sample size, the software retrieved the percent coefficient of variation [CV (%)] between calculated means. Calculated CV values in relation with statistical group size (n) were displayed in dot plots on *Y-axis* and *X-axis*, respectively. *Solid line* represents the selected CV threshold value and the *dotted line* represents the group statistical sample size (n = 16) required to maintain CV below the threshold for all analyzed growth factors

In this work, we assessed concentrations of selected growth factors in n = 44 SRGF specimens derived from aphaeretic PRP products taken from single donors (61% males and 39% females). Distribution of platelet concentrations in PRP products (squared box plot) as well as distribution of concentrations measured in single donor SRGF specimens (left box plots) are reported in Fig. 1. Below box plots on the left, mean growth factor concentrations together with CV values are reported. VEGF and FGF-b were characterized by the highest variability. We have applied our statistical algorithm to analyze the data matrix of growth factor concentrations measured in single donor SRGF. The algorithm was programmed to create in silico 500 groups of random data (single donor SRGF) and to repeat this operation increasing group statistical sample size step by step from n = 2 to n = 20. Thereafter, in created groups (n = 9500), the software calculated means for each growth factor. Finally, after matching groups characterized by the same statistical sample size, the software retrieved the CV between calculated means for each growth factor. Calculated CV values in relation with statistical group sample size are displayed in Fig. 2. In order to define a threshold, we estimated asymptotic CV values for all growth factors (VEGF: 19%, FGF-b: 13%, PDGF-AA: 6%, PDGF-AB: 5%, PDGF-BB: 7%, ES: 5%, AS: 7%, TGF-β1: 6%, IGF-I: 8% and EGF: 4%). For such reasons, we arbitrarily applied 20% as CV threshold to analyze all the growth factors (solid line in Fig. 2). When considering groups with a statistical sample size of n = 16, the CV between algorithm-calculated means of VEGF concentrations was below the 20% threshold. Considering all the other analyzed growth factors, the CV between mean concentration values calculated in groups of n = 16 statistical samples was far lower than 20%. Distribution of algorithm-calculated mean concentrations in groups characterized by a statistical sample size of n = 16 are reported in Fig. 1 (middle box plots). Grand mean of obtained means as well as CV values are reported below each middle box plots. For each growth factor, CV was strongly reduced when compared to single donor SRGF. To validate results obtained by mathematical algorithm, we prepared n = 10 pool product batches mixing together n = 16 randomly selected single donor SRGF product specimens. Table 1 lists the identification codes (from 1 to 44) of SRGF products actually mixed together to create the 10 different test pools (from A to J). Moreover, to demonstrate random SRGF selection for batch manufacturing, distribution of inclusion frequency of single donor SRGF specimens within produced batches was enclosed in Additional file 1: Figure S1. Data distribution was close to a normal Gaussian curve centered on the expected mean value of 3.64 times. Distribution of growth factor concentrations measured in pool product batches are reported in Fig. 1 (right box plots). Means of measured growth factor concentrations as well as CV values are reported below each right box plots: CV values were equal to or below the defined threshold. For each analyzed growth factor, no significant differences were demonstrated comparing mean concentration measured in single donor SRGF specimens with the grand mean of mean concentrations calculated in algorithm-created groups of data or with mean of measured concentrations in SRGF pool batches. Single donor concentration values measured by immunoenzyme assay were a posteriori grouped, considering exactly the composition of the real pool batches randomly created for approach validation (Table 1): means were calculated for each growth factor. These calculated means were not statistically different from related values measured in real pools by immunoenzyme assay (data not shown).

**Table 1:** Composition of manufactured supernatant rich in growth factors pool batches. Identification matrix of single donor supernatant rich in growth factors (n = 44) utilized used to create the n = 10 supernatant rich in growth factors pool batches (from A to J, see “Methods”). To produce SRGF pools batches A and B, 2 ml of single donor SRGF specimens were mixed together. Otherwise, to create SRGF pool batches C–J, only 500 μl of each single donor SRGF specimens were utilized

Batch A	Batch B	Batch C	Batch D	Batch E	Batch F	Batch G	Batch H	Batch I	Batch J
1	3	1	2	1	1	2	2	2	4
8	9	6	4	2	5	3	3	3	5
11	14	8	6	4	7	7	8	4	6
13	15	10	7	7	11	10	9	5	8
14	16	13	11	8	12	11	13	9	9
15	18	20	16	10	15	12	14	12	16
16	20	21	21	27	18	19	16	15	17
17	24	22	22	28	22	20	17	19	19
21	25	25	23	30	26	21	19	23	23
25	26	27	24	31	27	23	20	34	27
29	27	30	26	36	29	24	22	35	29
33	28	31	31	38	32	29	28	36	32
34	33	32	33	39	35	30	30	37	33
38	36	34	36	40	39	31	32	40	37
43	40	35	42	43	42	41	37	41	39
44	41	39	43	44	43	43	44	44	42

**Fig. 3:**
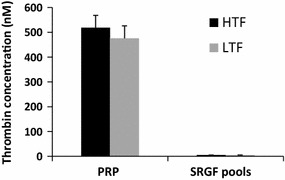
Concentrations of generated thrombin when low recombinant tissue factor (LTF) or high recombinant tissue factor (HTF) amounts were added to samples of platelet rich plasma (PRP) from platelet apheresis product (as control) or to samples of SRGF pool batches comprising n = 16 single donor specimens. Thrombin availability in SRGF was demonstrated to be undetectable

To further characterize SRGF products, we assayed residual thrombin availability in SRGF pool batches. Thrombin concentrations were measured also in freshly obtained PRP samples (as control). As shown in Fig. 3, thrombin availability in SRGF pools was almost undetectable.

**Fig. 4:**
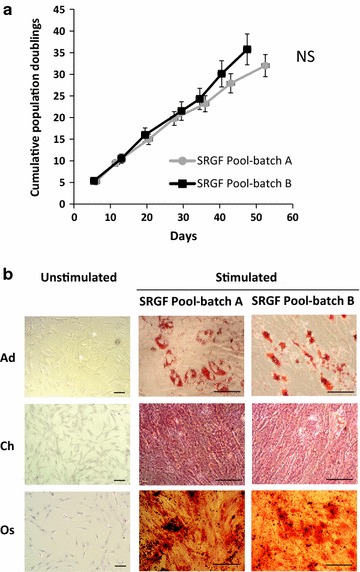
**a** Cumulative population doublings of ASC expanded by cell culture media containing 5% (vol/vol) SRGF pool—batch A or SRGF pool—batch B. ASC were derived from n = 2 separate patients and were both expanded taking advantage of each different culture medium. No statistical differences were observed. Data of each curve are presented as means of cPD characterizing ASC from both patients. *Error bars* represents SD. *Dots* in the graph represent cell passages. **b** Images of induced (Stimulated) and non induced (Unstimulated) differentiation into adipogenic (Ad), chondrogenic (Ch) and osteogenic (Os) lineages of ASC expanded in presence of 5% (vol/vol) SRGF pool—batch A or SRGF pool—batch B. Differentiated cells were stained by Oil Red-O, Safranin-O and Alizarin Red, respectively

To directly validate consistency between manufactured SRGF pool batches in terms of ability to stimulate cell growth, we expanded ASC derived from 2 different donors both in presence of SRGF pool—batch A or B. As shown in Fig. 4a, expansion period (from passage 1 to passage 7) lasted for 55 days and PD increases were linear and constant through the whole experimental period (R^2^ linearity test for both SRGF pool—batch A and batch B = 0.99). No significant slope differences (slope SRGF pool—batch A = 0.6; slope SRGF pool—batch B = 0.7; p = 0.89) was demonstrated when comparing growth curves. Mean PDT of ASC expanded in presence of SRGF pool—batches A and B were 34.2 ± 4.8 and 39.1 ± 5.5 h, respectively.

As displayed in Fig. 4b, ASC expanded in presence of both SRGF pool—batch A and B efficiently differentiated into adipogenic (Ad), chondrogenic (Ch) and osteogenic (Os) lineages upon appropriate stimulation in cell culture.

## Discussion

Fetal bovine serum is the most commonly used source of growth factor to promote cell growth in culture. When cell products are intended for utilization as an ATMP, GMP guidelines must be fulfilled [2, 21]. Beside environmental safety requirements, characteristics of consumables and additives utilized for cell expansion are regulated by GMP guidelines [1]. All products employed within the cell expansion process must be of non-animal origin and different product batches must be sufficiently standardized in order to allow the achievement of safe cell products with consistent yield and quality. In previous works, we described the production protocol and growth factor content of our human platelet derived SRGF product [15], and we demonstrated that SRGF can improve stem cell growth rate in culture [22]. Thus, we considered SRGF as a promising choice as GMP compliant cell culture additive. Nevertheless, inter-individual variability in growth factor concentrations was demonstrated when considering growth factor mixtures obtained from platelet single donors [15]. Overcoming this drawback is a requirement for a safe and acceptable SRGF utilization in GMP production processes. Pooling together different SRGF single donor products can significantly improve consistency of growth factor concentrations in final product batches. Little is known about the adequate number of single donor SRGF to be pooled together in order to obtain consistent final products characterized by the lowest possible variability. Direct growth factor concentration testing in pools of single donor SRGF products with different pool size would be excessively expensive and time consuming. Thus, we assayed selected growth factor concentrations in n = 44 single donor SRGF specimens. Thereafter, to predict an adequate pool statistical sample size utmost reducing variability between final product batches, we applied a customized statistical algorithm to the data matrix of single donor concentrations. The statistical algorithm was based on the assumption that a calculated mean of single donor SRGF concentration values (separately measured) could closely represent the real concentration value in a pool comprising equal volumes of the same SRGF specimens. Our algorithm simulated in silico the production of several SRGF pools characterized by growing sample sizes and, in turn, estimated the CV of putatively obtained final concentrations (means). We conclude that pooling together equal volumes of n = 16 single donor SRGF products can minimize the variability of growth factor content in obtained pools. This limiting pool size value was determined by the analysis of the most variable growth factor in our results, i.e. VEGF. The small number (n = 44) of analyzed single donor products was due to technical and practical reasons; in the analysis performed by the algorithm, this limited number probably determined repeated selection of the same single donor data, especially when considering higher sample size groups. Thereafter, we attempted to validate our in silico obtained results directly analyzing growth factor concentrations in SRGF pool batches, each comprising equal volumes of n = 16 single donor SRGF specimens. Again, limited availability of different single donor SRGF prompted us to validate our approach considering the same samples used to obtain the data matrix analyzed by the statistical algorithm. Growth factor concentrations, calculated by the algorithm averaging groups of concentration values measured in single donor SRGF products, were not significantly different from actual values measured in real pool batches. This evidence confirms that theoretical assumptions characterizing our statistical algorithm were acceptable. As evidenced by box plots and considering CV changes, a strong and effective improvement of final product quality was demonstrated for concentration values measured by immunoenzyme assay in pool batches of n = 16 single SRGF products. In particular, CV values of concentrations actually measured in the analyzed pool batches were all equal or below the 20% threshold determined by our statistical algorithm. Furthermore, we aimed to demonstrate consistency of biological activity in cell culture of our SRGF pool batches. Thus, we expanded ASC obtained from 2 different patients in separate culture conditions, i.e. utilizing SRGF pool—batch A and SRGF pool—batch B as growth medium supplements. The impact of different SRGF concentrations in cell culture medium was previously described [22] and 5% (vol/vol) concentration was chosen to standardize ASC expansion protocol. By analyzing cell growth curve we demonstrated that, in both conditions, PDN increased constantly throughout the study. Moreover, we showed that growth rates of expanded ASC in media containing different SRGF pool batches where closely similar. In addition, we demonstrated that different SRGF pool batches as medium additive failed to differently affect adipogenic, osteogenic and chondrogenic differentiation potential of expanded ASC. Thus, we can conclude that medium additives, each obtained pooling together at least n = 16 single donor SRGF specimens, consistently modulated pivotal features of cultured ASC, as proliferation rate and differentiation potency. The graphic analysis obtained by the statistical algorithm suggested that increasing to over n = 16 the number of single donor SRGF in pools can reduce variability of growth factor concentration between batches. Nevertheless, we are confident that such potential improvement in the final product quality would not be relevant in cell culture applications, at least in terms of biological activity consistency between batches. Thus, we can suggest that utilization of medium supplements manufactured pooling together larger cohorts of single donor SRGF specimens would not further improve consistency of final product biological activity. In previous reports, the optimal number of single donor platelet concentrates to be mixed together for standardization of the final products was reported to be higher [14] or in line [13] with our results. In this work, we suggest the limiting number of single donor SRGF needed to manufacture a standardized medium additive for academic applications, applying a predictive statistical analysis and in turn validating results in biological samples and in cell culture applications. Distributions of platelet apheresis donor age (within 18–60 years, as regulated by Italian laws and “Centro Nazionale Sangue” of the Italian Ministry of Health) and gender can exclude direct donor-related influence on growth factor concentration in the final SRGF pool product [23, 24]. In parallel, we showed that our SRGF is substantially thrombin free, which confers an additional added value to the product as stromal cells express thrombin-specific receptors [25]. In addition, a previous work [26] has demonstrated that thrombin stimulates proliferation of fibroblasts. Cell expansion by platelet lysates requires heparin addition to inhibit the thrombin dependent coagulation cascade [9] and heparin can affect proliferation of stromal cells in culture [27, 28]. Consequently, as heparin was not used in our experimental conditions, we can exclude the non-specific influence of both thrombin and heparin on observed ASC proliferation.

## Conclusions

We demonstrated that pooling together equal volumes of n = 16 single donor SRGF specimens to obtain a final product batch, can strongly decrease variability in growth factor content. Consistency between pool product batches was demonstrated with regard to: (a) concentrations of selected growth factors; (b) the potential to stimulate ASC expansion in culture; and (c) the potential to induce ASC differentiation into adipogenic, osteogenic and chondrogenic lineages. Results obtained by our statistical approach suggested that increasing the pool size of single donor SRGF product would not provide any further improvement in terms of biological activity consistency of final SRGF pool batches.

## Additional file

**Additional file 1: Figure S1.** Graphical representation of the distribution of single donor SRGF inclusion frequency (times) in randomly created n = 10 (from A to J) test batches. Data distribution is close to a normal Gaussian curve centered on the expected mean value of 3.64 times.

## Abbreviations

SRGF: supernatant rich in growth factors
ATMP: advanced therapy medicinal products
GMP: good manufacturing practice
ASC: adipose tissue mesenchymal stromal/stem cells
VEGF: vascular endothelial growth factor
EGF: epidermal growth factor
PDGF-AA, AB, -BB: platelet derived growth factor-AA, -AB, -BB
TGF-β1: transforming growth factor-β1
FGF-basic: fibroblast growth factor basic
IGF-I: insulin like growth gactor-I
ES: endostatin
AS: angiostatin
LTF: low concentrations of recombinant tissue factor
HTF: high concentrations of recombinant tissue factor
PRP: platelet rich plasma
CV: coefficient of variation

## References

[1] Expert Committee of European Commission. Good manufacturing practice—volume 4. The rules governing medicinal products in the European Union; 2011.

[2] Karnieli O, Friedner OM, Allickson JG, Zhang N, Jung S, Fiorentini D (2017). A consensus introduction to serum replacements and serum-free media for cellular therapies. Cytotherapy.

[3] Spees JL, Gregory CA, Singh H, Tucker HA, Peister A, Lynch PJ (2004). Internalized antigens must be removed to prepare hypoimmunogenic mesenchymal stem cells for cell and gene therapy. Mol Ther.

[4] Mackensen A, Drager R, Schlesier M, Mertelsmann R, Lindemann A (2000). Presence of IgE antibodies to bovine serum albumin in a patient developing anaphylaxis after vaccination with human peptide-pulsed dendritic cells. Cancer Immunol Immunother.

[5] Abbott A, Cyranoski D (2003). Biologists seek to head off future sources of infection. Nature.

[6] Hill AF, Desbruslais M, Joiner S, Sidle KC, Gowland I, Collinge J (1997). The same prion strain causes vCJD and BSE. Nature.

[7] Committeee for medicinal products for human use (CHMP). Note for guidance on the use of bovine serum in the manufacture of human biological products. European Medicine Agency; 2012. p. 1–8.

[8] WHO expert group. WHO guidelines on tissue infectivity distribution in transmissible spongiform encephalopaties. World Health Organization; 2010. p. 1–21.

[9] Hemeda H, Giebel B, Wagner W (2014). Evaluation of human platelet lysate versus fetal bovine serum for culture of mesenchymal stromal cells. Cytotherapy.

[10] Shih DT, Burnouf T (2015). Preparation, quality criteria, and properties of human blood platelet lysate supplements for ex vivo stem cell expansion. N Biotechnol.

[11] Capelli C, Pedrini O, Valgardsdottir R, Da RF, Golay J, Introna M (2015). Clinical grade expansion of MSCs. Immunol Lett.

[12] Doucet C, Ernou I, Zhang Y, Llense JR, Begot L, Holy X (2005). Platelet lysates promote mesenchymal stem cell expansion: a safety substitute for animal serum in cell-based therapy applications. J Cell Physiol.

[13] Schallmoser K, Strunk D (2013). Generation of a pool of human platelet lysate and efficient use in cell culture. Methods Mol Biol.

[14] Schallmoser K, Strunk D (2009). Preparation of pooled human platelet lysate (pHPL) as an efficient supplement for animal serum-free human stem cell cultures. J Vis Exp.

[15] Durante C, Agostini F, Abbruzzese L, Toffola RT, Zanolin S, Suine C (2013). Growth factor release from platelet concentrates: analytic quantification and characterization for clinical applications. Vox Sang.

[16] Araki J, Jona M, Eto H, Aoi N, Kato H, Suga H (2012). Optimized preparation method of platelet-concentrated plasma and noncoagulating platelet-derived factor concentrates: maximization of platelet concentration and removal of fibrinogen. Tissue Eng Part C Methods.

[17] Bernardi M, Albiero E, Alghisi A, Chieregato K, Lievore C, Madeo D (2013). Production of human platelet lysate by use of ultrasound for ex vivo expansion of human bone marrow-derived mesenchymal stromal cells. Cytotherapy.

[18] The R Project for statistical computing. Web. https://www.r-project.org. Accessed 21 Apr 2017.

[19] Efron B, Tibshirani RJ (1993). An introduction to the Bootstrap.

[20] Bourin P, Bunnell BA, Casteilla L, Dominici M, Katz AJ, March KL (2013). Stromal cells from the adipose tissue-derived stromal vascular fraction and culture expanded adipose tissue-derived stromal/stem cells: a joint statement of the International Federation for Adipose Therapeutics and Science (IFATS) and the International Society for Cellular Therapy (ISCT). Cytotherapy.

[21] Astori G, Amati E, Bambi F, Bernardi M, Chieregato K, Schafer R (2016). Platelet lysate as a substitute for animal serum for the ex vivo expansion of mesenchymal stem/stromal cells: present and future. Stem Cell Res Ther.

[22] Borghese C, Agostini F, Durante C, Colombatti A, Mazzucato M, Aldinucci D (2016). Clinical-grade quality platelet-rich plasma releasate (PRP-R/SRGF) from CaCl2 -activated platelet concentrates promoted expansion of mesenchymal stromal cells. Vox Sang.

[23] Weibrich G, Kleis WK, Hafner G, Hitzler WE (2002). Growth factor levels in platelet-rich plasma and correlations with donor age, sex, and platelet count. J Craniomaxillofac Surg.

[24] Lohmann M, Walenda G, Hemeda H, Joussen S, Drescher W, Jockenhoevel S (2012). Donor age of human platelet lysate affects proliferation and differentiation of mesenchymal stem cells. PLoS ONE.

[25] Chen J, Ma Y, Wang Z, Wang H, Wang L, Xiao F (2014). Thrombin promotes fibronectin secretion by bone marrow mesenchymal stem cells via the protease-activated receptor mediated signalling pathways. Stem Cell Res Ther.

[26] Zhou S, Xiao W, Pan X, Zhu M, Yang Z, Zhang F (2014). Thrombin promotes proliferation of human lung fibroblasts via protease activated receptor-1-dependent and NF-kappaB-independent pathways. Cell Biol Int.

[27] Dombrowski C, Song SJ, Chuan P, Lim X, Susanto E, Sawyer AA (2009). Heparan sulfate mediates the proliferation and differentiation of rat mesenchymal stem cells. Stem Cells Dev.

[28] Hemeda H, Kalz J, Walenda G, Lohmann M, Wagner W (2013). Heparin concentration is critical for cell culture with human platelet lysate. Cytotherapy.

