# Supplementary material for: Standardization of platelet releasate products for clinical applications in cell therapy: a mathematical approach
Source: J Transl Med. 2017 May 19;15:107. doi: 10.1186/s12967-017-1210-z (PMC5437585; doi:10.1186/s12967-017-1210-z)
Supplement: Supplementary file 1 — Additional file 1: Figure S1. Graphical representation of the distribution of single donor SRGF inclusion frequency (times) in randomly created n = 10 (from A to J) test batches. Data distribution is close to a normal Gaussian curve centered on the expected mean value of 3.64 times. [file 12967_2017_1210_MOESM1_ESM.pptx]

## Slide 1
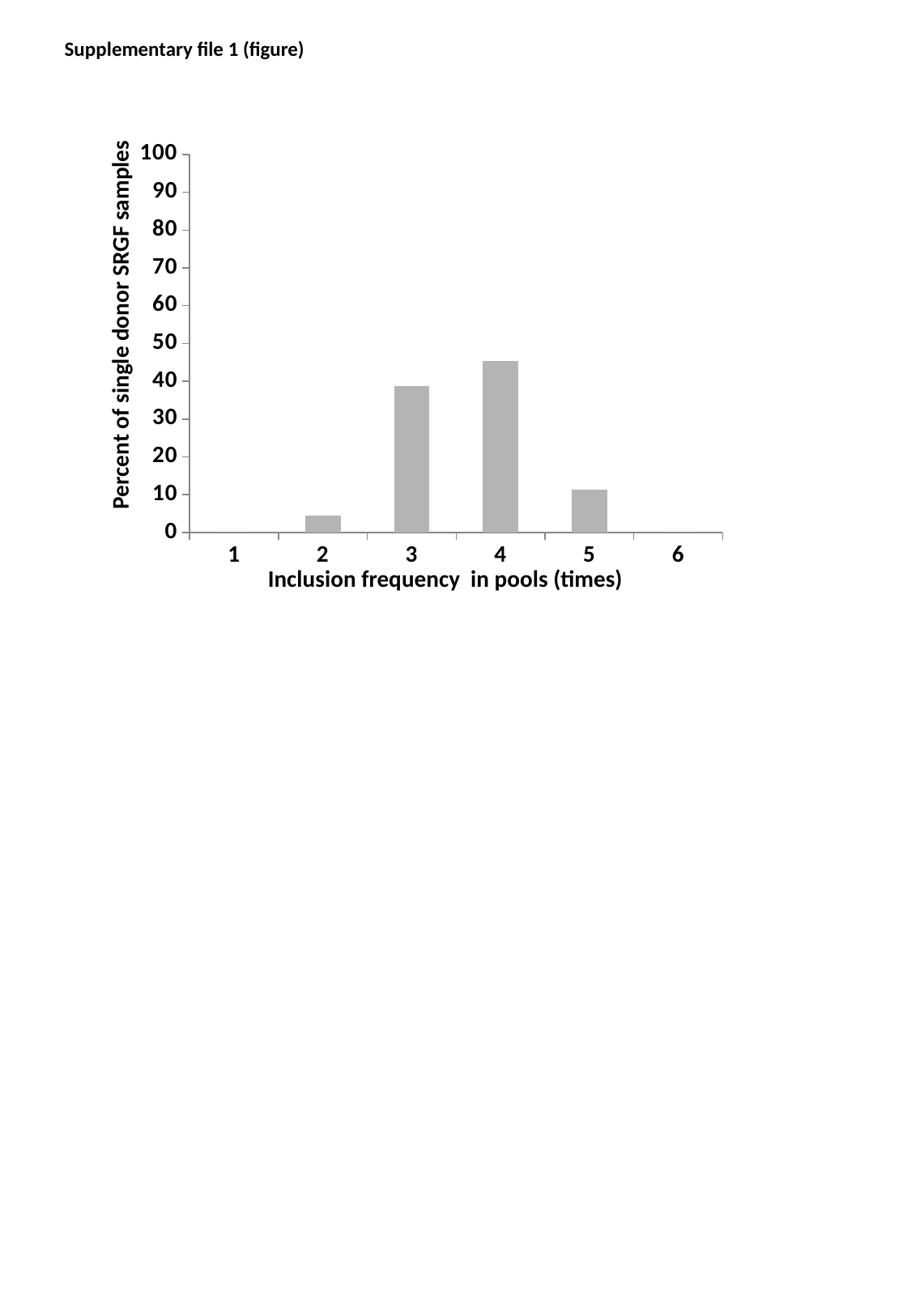

Supplementary file 1 (figure)
### Chart
| Category | |
|---|---|Percent of single donor SRGF samples
Inclusion frequency in pools (times)
